# Evaluation of the lights4violence program: reduction in machismo and acceptance of violence among adolescents in Europe

**DOI:** 10.1186/s12889-022-12770-4

**Published:** 2022-03-03

**Authors:** Vanesa Pérez-Martínez, Belén Sanz-Barbero, Rosario Ferrer-Cascales, Nicola Bowes, Alba Ayala, Miriam Sánchez-SanSegundo, Natalia Albaladejo-Blázquez, Nicoletta Rosati, Sofia Neves, Cristina Pereira Vieira, Barbara Jankowiak, Sylwia Jaskulska, Katarzyna Waszyńska, Carmen Vives-Cases

**Affiliations:** 1 Community Nursing, Preventive Medicine and Public Health and History of Science Department, University of Alicante, Alicante, Spain; 2 CIBER of Epidemiology and Public Health (CIBERESP), Madrid, Spain; 3 National School of Public Health, Carlos III Health Institute, Madrid, Spain; 4 Health Psychology Department, University of Alicante, San Vicente del Raspeig, Alicante, Spain; 5 Cardiff School of Sport and Health Sciences, Cardiff Metropolitan University, Cardiff, UK; 6 Department of Statistics, University Carlos III of Madrid, Madrid, Spain; 7 Department of Human Sciences, LUMSA-Libera Universita Maria SS Assunta Di Roma, Rome, Italy; 8 University of Maia, Maia, Portugal; 9 CIEG (ISCSP-Ulisbon) PT Best, Lisbon, Portugal; 10 Faculty of Educational Studies, Adam Mickiewicz University, Poznan, Poland

**Keywords:** Machismo, Acceptance of violence, Empathy, Adolescents, Intervention, Intimate partner violence

## Abstract

**Background:**

Machismo and acceptance of violence (AV) against women are part of the social construction of hegemonic masculinity and are related to the risk of dating violence. This study aims to analyze the effectiveness of the Lights4Violence program in reducing machismo and AV in secondary school students from different European cities.

**Methods:**

Quasi-experimental longitudinal study using a convenience sample of 1,146 high school students from different European cities (12–17 years old) including 575 intervention group students (59.1% girls) and 571 control group students (62.7% girls). We performed linear regression models to identify the effect of the intervention, modelling the difference in means in machismo and AV (dependent variables) between wave-2 and wave-1.

**Results:**

An interaction was identified between the group variable and the empathy variable. In wave-2, girls with high empathy at baseline in the intervention group obtained lower mean AV scores (β: -0.131; *p* = 0.004). However, the boys in the intervention group (reference: control group) with low empathy at baseline registered a significant increase in the mean values of machismo (β: 0.247; *p* < 0.001).

**Conclusion:**

The importance of empathy is shown in the effectiveness of interventions to reduce machismo and AV in adolescents. While the Lights4Violence program focuses on promoting healthy relationships, there were some controversial results. It is possible that some children, especially those with less empathy, may have felt “challenged” during the intervention and/or assessment. This suggests the need for the development of interventions that also consider psychological processes and integrate the promotion of positive expressions of masculinity.

## Background

Intimate partner violence (IPV) among adolescents is a serious public health problem and a violation of human rights that affects society in its entirety at the global level. Its negative effects on health are widely documented in scientific literature, which shows that people and their environments are negatively influenced over the medium/long-term [1]. In fact, not only is IPV an expressive phenomenon in statistical terms, it has an impact on victims’ well-being, quality of life and overall life satisfaction. In the European Union countries, 43% of women have experienced some form of psychological violence in their relationships since the age of 15, and one in five women (22%) have experienced physical and/or sexual IPV [2]. In addition, 22% have been a victimized by someone other than their partner [2]. Young women have reported higher levels of IPV victimization in the past 12 months, compared to older women (6% vs 3–5%) [2]. This finding is consistent internationally [1].

Experiencing IPV is associated with significant increase in the risk of developing physical injuries, chronic diseases, psychopathology and suicidal ideation [3]. Moreover, victims of IPV show poorer levels of performance in several areas of functioning and lower levels of social integration when compared with non-victims [4].

The prevention of this problem requires integrating work on masculine gender norms, and therefore macho attitudes, given the relationship with violent behavior [5]. Machismo is defined as “the embarrassment over backing down, and justification of violence in response to threat and attack, or violence as a part of being male and strong, and weakness associated with fear and non-violence” [6]. It is expressed through macho attitudes that highlight the domination of men over women through different behaviours [5]. Machismo and acceptance of violence (AV) against women is a part of the social construction of hegemonic masculinity, which is shared by many, and suffered by those who do not share it [6]. This perception of macho culture affects not only boys, but is also internalized by girls, who normalize and assume discriminatory gender roles [7].

Studies have shown that there is a relationship between victimization by IPV and higher levels of machismo and AV, both in boys and girls [8]. Longitudinal results suggest that the traditional attitudes associated with gender roles occur among boys with high levels of AV during dating [5]. Gendered social relations, and particularly gender roles, influence IPV [9]. Research has indicated that conservative gender norms are linked to increases in the likelihood of perpetrating and experiencing IPV [10]. In terms of victims of IPV, some studies show that high levels of acceptance of emotional and physical abuse increases the risk of exposure to these forms of violence in their partner relationships [11].

Personal competencies such as empathy can positively influence the development of partner relationships, but they can also influence the risk of IPV when there are deficits [12]. Both the lack of empathy and AV are precursors to violent conduct in heterosexual men [13]. On the other hand, health assets, such as the social support (SS) of parents, can have a protective effect against IPV [14]. However, the lack of an appropriate role model in parents regarding confrontation and anger management can also influence how girls treat others and how they are treated by others [15].

School-based programs to prevent or reduce dating violence behaviours or attitudes in adolescents have obtained different results by sex [16]. Although there are no interventions that have studied the interaction of empathy in the effectiveness of reducing dating violence attitudes, it has been found that empathy acts as a mediator in the association between risk factors (i.e., family violence victimization) and dating violence perpetration, especially in boys [17], and victimization in girls [12]. This highlights the importance of training in empathy to prevent this type of violence [17]. Similar results have been obtained with respect to aggressiveness and social support from parents [18]. Witnessing negative conflict resolution strategies in parents could increase aggressiveness, leading to a higher probability of perpetrating DV [18]. The levels of empathy, aggressiveness and the social support from parents could have an influence on modifying certain attitudes and behaviors.

### The lights4violence project

This study was based on the European Project “Lights, Camera and Action against Dating Violence” (Lights4Violence), carried out during the 2017–2019 period with the objective of promoting protective assets (personal competencies and external resources) to support the development of healthy relationships among secondary school youth [19]. It was based on the positive youth development model and focused on individual, family, and community efforts to improve and strengthen health [20]. The project was carried out in six European cities: Alicante, Rome, Iasi, Matosinhos, Poznan and Cardiff.

The intervention was made up of five modules, each of which included between 15 and 17 sessions of approximately 50-min. The first module concerned personal assets and external assets for positive development and the promotion of healthy couple relationships, as well as key concepts related to gender and violence. The second module concerned competencies that promote healthy couple relationships (communication abilities, empathy, prosocial skills, etc.) The third, fourth and fifth modules were focused on learning about cinema and film. Students were asked to develop a short film that would exemplify the knowledge they had acquired during the program [21]. The intervention was carried out by technicians with training in psychology and researchers of the project, with the involvement of the teaching staff, to ensure the fidelity of the intervention during the pilot study [20].

The research questions were as follows:

1. Has the intervention changed the levels of machismo and acceptance of violence in boys and/or girls?
2. What influence do baseline scores in empathy, aggressiveness and/or social support have on intervention’s result?

Based on these questions the objective of the research was to analyze the effects of the Lights4Violence intervention on machismo and AV in secondary school students from different European cities by sex and baseline social support, aggressiveness, and empathy in comparison with peers enrolled in selected control schools.

## Methods

Quasi-experimental, longitudinal study with a convenience sample of secondary school students (ranging in age from 12–17) [20].

### Participants

We recruited 1,146 students in Alicante, Spain (*n* = 176, 53.9% girls), Rome, Italy (*n* = 241, 71.4% girls), Iasi, Romania (*n* = 253, 62.0% girls), Matosinhos, Portugal (*n* = 210, 51.4% girls), Poznan, Poland (*n* = 109, 69.7% girls) and Cardiff, United Kingdom (*n* = 166, 54.2% girls). An analysis of statistical power was carried out in order to estimate the sample size (sample size designed for 1,300 students), based on data from a previously published meta-analysis of 23 studies on school interventions that aimed to prevent violence and negative couple attitudes among adolescents [16]. The data were collected from 12 schools between October 2018 and June 2019. An online, multi-language questionnaire was used in the participating schools. It was validated using back-translation, which involves re-translating the questionnaire from one language back to its original language and comparing the two for consistency. The schools assigned to the control group had the same composition in terms of age, sex, and academic course as the schools assigned to the intervention group. In both groups, schools were selected with a similar socioeconomic level (in terms of the social characteristics and location of the school). The questionnaire was completed both by the intervention group as well as the control group, prior to beginning the intervention (wave 1) and after the intervention (wave 2). The control group did not receive the intervention, but data were collected at the same time as the intervention group. Given the analysis was stratified by sex, 0.6% of the cases were eliminated due to responses of “others” in the sex variable. Around 74.2% participated in wave 2 compared to wave 1.

### Variables

#### Machismo and AV

The Maudsley Violence Questionnaire (MVQ) was used to assess machismo and AV [7]. It is composed of 56 items (true–false scale) that represent norms and beliefs that justify and support violence. It is made up of two subscales: “machismo” (42 items; 0–42 range) and “acceptance of violence” (14 items; 0–14 range). Cronbach’s Alpha was 0.914 for the machismo subscale in boys and 0.861 for girls. For the acceptance of violence subscale, the Crobach’s Alpha was 0.755 for boys and 0.728 for girls [6]. This scale has no cut-off point, so the higher score represents greater machismo and acceptance of violence.

#### Other variables

- Sociodemographic characteristics: age, sex and fathers/mothers education level. Responses were collected using a multiple-choice format. For education variables, the option “secondary/higher” included the following categories: complete secondary (mandatory secondary education, high school or first level technical school) and complete university (associates degree, certificate, bachelor's degree or second level of technical school).
- Exposure to IPV [22]: Information was collected on having a partner and exposure to IPV. Later, these were combined into “physical and/or sexual violence” and “fear/control” variables. Then the “experience with partner violence” variable was defined, with three response categories: those who had never had a partner relationship, those who had been in a relationship and were victims of IPV (physical, sexual, fear/control IPV) and those who had had a partner relationship but were not victims of IPV. Items were previously described in detail elsewhere [20].
- Child and Adolescent Social Support Scale [23]: Items were previously described in detail elsewhere [20]. For this study, only the frequency subscale was analyzed from parents’ and teachers’ subscales (12–72 range for each area), because the association in both dimensions with the dependent variables and covariables was very similar. The five response options for the dimensions made up a Likert type scale ranging from “totally disagree” to “totally agree”. Cronbach’s Alpha was 0.94 for the total and varied from 0.87 to 0.93 in the four subscales. For parents’ social support, good social support is considered when scores are over 54, and for teacher’s social support, when scores are over 50.
- Aggression Questionnaire-Refined [24]: The whole questionnaire was previously described in detail elsewhere [20]. We used the 12-item version that uses a Likert type scale with five options ranging from 1 (never) to 5 (always). Cronbach’s Alpha between factors ranged from 0.70 to 0.83. There is considered to be high aggressiveness when scores are higher than 27.
- Empathy: Measured using three items related to the capacity to feel and understand the emotions and ways of thinking of others [25]: “I can tell what other people are feeling”, “I am able to tell when other people are upset” and “I can understand another person’s way of thinking”. All of the items used a Likert type scale with four scores ranging from “strongly disagree” to “strongly agree”. Cronbach’s Alpha ranged from 0.71 to 0.77. Given that for our study three items were used from the Personal Strengths Inventory-2, which assesses personal strengths related to socio-emotional competences, a reliability test was carried out (Cronbach’s Alpha 0.680). Empathy is considered high when the scores are higher than 8.

### Ethical considerations

Data was collected by project partners based at universities in the various countries. The data was collected and stored anonymously, and participants created a unique participant code for themselves during the first data collection point. Participation was voluntary, and each partner university was required to obtain the permission of their own ethical committees. Schools provided a signed informed consent document from the school directors, as did parents of the participants and the students themselves.

The Lights4Violence protocol was approved by the ethical committee of each university. It was also registered in ClinicalTrials.gov by the coordinator (Clinicaltrials.gov: NCT03411564. Unique Protocol ID: 776905. Date registered: 18-01-2018).

### Data analysis

A descriptive analysis was carried out to describe the sample in wave-1 for the control and intervention groups, identifying differences by sex, using chi square differences for categorical variables (Table 1). The Cohen’s d effect size [26] was calculated based on the differences between the means in machismo and AV, obtained from the intervention and control groups. Mean and standard deviations were obtained- using paired student t tests- for the subscales of machismo and AV, sociodemographic variables, violence variables, SS, aggressiveness, and empathy, to identify differences by sex and group across time (Tables 2 and 3). Linear regression models were used to identify the intervention effect, by modeling the difference in means of machismo and AV (dependent variables), between wave-2 and wave-1 (Table 4). First, a crude analysis was carried out, and later variables were introduced one by one to ensure contribution to the model (stepwise forward). The difference in the value obtained in wave-2 and wave-1 was the result variable $\left({Y}_{i2}-{Y}_{i1}\right)$, where ${Y}_{i2}$ is the observation for student i in wave-2 and ${Y}_{i1}$ is the observation for student i in wave-1 (Eq. 1). The intervention effect was identified by the group variable (control/intervention). Models were adjusted by the following covariables: baseline result value $\left({Y}_{i1}\right)$, city, age, mother’s education, and the following scales in wave-1: empathy, SS of fathers/mothers and teachers (CASSS) and aggressiveness (AQR).

**Table 1:** Description of the Sample in Wave 1, Lights4Violence Project

	Girls					Boys				
	Control		Intervention		*p*-value	Control		Intervention		*p*-value
	N	%	N	%		N	%	N	%	
Age					0,005*					*P* < .001*
< = 13 years	94	26.3	123	36.2		66	31	112	47.7	
> 13 years	264	73.7	217	63.8		147	69	123	52.3	
Mother's education					0.031*					0.176
Primary and lower	34	9.6	51	15		26	12.4	40	17	
secondary/superior	319	90.4	289	85		183	87.6	195	83	
Missings	5	1.4	0	0		4	1.9	0	0	
Dating violence (n.a = 12)					0.001*					0.105
I have never been in a dating relationship	171	47.8	116	34.5		80	37.6	64	28.2	
Yes	67	18.7	69	20.5		36	16.9	41	18.1	
No	120	33.5	151	44.9		97	45.5	122	53.7	
Parents social support (range; 12, 72)					0.800					0,091
< = 54	184	51.4	178	52.4		103	48.4	95	40.4	
> 54	174	48.6	162	47.6		110	51.6	140	59.6	
Teachers social support (range; 12, 72)					0.029*					0.268
< = 50	196	54.7	158	46.5		99	46.5	97	41.3	
> 50	162	45.3	182	53,5		114	53,5	138	58.7	
Aggressiveness (range; 12. 60)					0.189					0.079
< = 27	191	53.4	162	48.4		92	43.2	120	51.5	
> 27	167	46.6	173	51.6		121	56.8	113	48.5	
Missings	0	0	5	1.5		0	0	2	0.9	
Empathy (range; 13. 100)					0.857					0.224
< = 8	126	35.2	120	36.3		96	45.1	91	39.6	
> 8	228	63.7	211	63.7		116	54.5	139	60.4	
Missings	4	1.1	9	2.6		1	0.5	5	2.1	

^*^p < 0.05

**Table 2:** Mean and standard deviations of machismo for sociodemographic and violence variables at baseline

	Girls						Boys					
	Control			Intervention			Control			Intervention		
	Pre-test	Post-test		Pre-test	Post-test		Pre-test	Post-test		Pre-test	Post-test	
	Mean (SD)	Mean (SD)	*p*-value	Mean (SD)	Mean (SD)	*p*-value	Mean (SD)	Mean (SD)	*p*-value	Mean (SD)	Mean (SD)	*p*-value
Age												
< = 13 years	5.3 (5.9)	4.4 (4.5)	0.10	5.4 (5.8)	5.8 (6.6)	0.49	8.6 (7.3)	7.0 (6.5)	0.06	8.7 (8.2)	9.3 (8.4)	0.44
> 13 years	5.8 (5.8)	6.1 (5.8)	0.73	6.5 (5.8)	6.5 (6.8)	0.81	12.0 (8.8)	11.2 (8.2)	0.19	11.2 (7.9)	11.2 (8.9)	0.98
Mother’s education												
Primary and lower	7.8 (8.4)	6.3 (7.3)	0.29	8.7 (7.8)	8.7 (8.9)	0.97	15.0 (10.6)	13.0 (8.3)	0.24	12.9 (9.6)	12.5 (11.2)	0.81
Secondary-superior	5.5 (5.3)	5.5 (3.3)	0.85	5.6 (5.3)	5.9 (6.2)	0.47	10.4 (8.0)	9.6 (7.8)	0.08	9.5 (7.7)	9.9 (8.0)	0.32
Dating violence experience												
I have never been in a dating relationship	4.7 (4.5)	4.8 (4.6)	0.84	5.1 (5.3)	5.7 (6.5)	0.21	8.6 (7.8)	7.5 (7.0)	0.08	8.6 (6.6)	9.8 (8.2)	0.23
Yes	7.7 (6.6)	7.7 (7.3)	1	8.6 (7.2)	8.2 (7.9)	0.69	15.4 (8.6)	12.1 (6.8)	0.019*	13.1 (8.6)	12.1 (8.4)	0.23
No	6.0 (6.3)	5.4 (5.3)	0.31	5.6 (5.1)	5.9 (6.3)	0.60	11.2 (8.3)	11.1 (8.5)	0.93	10.1 (8.6)	10.2 (9.1)	0.84
Parents social support												
< = 54	6.6 (5.8)	6.2 (6.0)	0.34	7.1 (6.3)	7.2 (7.0)	0.75	11.5 (8.0)	11.4 (8.0)	0.80	11.6 (8.1)	11.5 (8.6)	0.82
> 54	4.8 (5.3)	4.8 (4.9)	0.83	5.0 (5.0)	5.3 (6.3)	0.56	10.4 (8.9)	8.6 (7.6)	0.017*	8.9 (7.9)	9.5 (8.7)	0.32
Teachers social support												
Low < = 50	6.0 (5.8)	5.8 (5.9)	0.67	6.8 (6.4)	6.6 (7.0)	0.71	11.8 (8.2)	10.5 (7.7)	0.12	12.4 (8.9)	11.0 (8.5)	0.07
High > 50	5.3 (5.5)	5.2 (5.1)	0.75	5.5 (5.2)	6.0 (6.5)	0.81	10.2 (8.7)	9.4 (8.0)	0.16	8.4 (7.1)	9.9 (8.8)	0.01*
Aggression Questionnaire Refined												
Low < = 27	4.0 (3.8)	4.2 (4.4)	0.55	3.7 (3.6)	4.3 (4.6)	0.09	7.7 (8.1)	6.9 (7.1)	0.27	6.5 (5.8)	7.4 (7.3)	0.21
High > 27	7.6 (6.8)	7.1 (6.2)	0.32	8.2 (6.5)	8.0 (7.8)	0.83	13.4 (7.9)	12.2 (7.7)	0.068	13.7 (8.6)	13.4 (9.0)	0.69
Empathy												
Low < = 8	6.0 (5.1)	5.7 (5.6)	0.52	6.5 (6.2)	7.2 (7.0)	0.22	11.3 (8.8)	9.4 (7.6)	0.025*	11.0 (8.6)	11.4 (8.3)	0.65
High > 8	5.6 (6.0)	5.5 (5.5)	0.83	5.7 (5.5)	5.6 (6.4)	0.77	10.7 (8.3)	10.4 (8.2)	0.63	9.5 (7.8)	9.7 (9.0)	0.72
Total	5.70 (5.66)	5.55 (5.50)	0.598	6.06 (5.79)	6.27 (6.74)	0.527	10.94 (8.47)	9.92 (7.9)	0.036*	10.02 (8.1)	10.31 (8.67)	0.528

^*^p < 0.05

**Table 3:** Mean and standard deviations of acceptance of violence for sociodemographic and violence variables at baseline

	Girls						Boys					
	Control			Intervention			Control			Intervention		
	Pre-test	Post-test		Pre-test	Post-test		Pre-test	Post-test		Pre-test	Post-test	
	Mean (SD)	Mean (SD)	*p*-value	Mean (SD)	Mean (SD)	*p*-value	Mean (SD)	Mean (SD)	*p*-value	Mean (SD)	Mean (SD)	*p*-value
Age												
< = 13 years	4.2 (3.1)	5.6 (3.3)	< 0.001*	4.3 (2.9)	5.0 (3.3)	0.026*	7.0 (3.5)	7.1 (3.0)	0.69	7.2 (3.3)	8.0 (3.0)	0.11
> 13 years	4.2 (3.1)	4.8 (3.5)	< 0.001*	4.0 (3.0)	4.4 (3.4)	0.019*	7.6 (3.2)	8.0 (3.2)	0.09	7.3 (3.1)	7.6 (3.1)	0.35
Mother’s education												
Primary and lower	3.3 (2.7)	3.8 (3.2)	0.33	4.8 (3.3)	5.1 (3.8)	0.64	7.0 (2.6)	7.1 (2.8)	0.81	7.3 (2.9)	7.6 (2.8)	0.57
Secondary-superior	4.3 (3.1)	5.1 (3.5)	< 0.001*	4.0 (2.9)	4.5 (3.3)	0.001*	7.6 (3.4)	7.9 (3.2)	0.11	7.5 (3.2)	7.8 (3.1)	0.089
Dating violence experience												
I have never been in a dating relationship	3.8 (2.9)	4.7 (3.3)	< 0.001*	3.5 (2.7)	4.1 (3.3)	0.021*	6.8 (3.6)	7.1 (3.6)	0.23	6.7 (3.7)	7.9 (3.5)	0.002*
Yes	5.0 (3.2)	5.6 (3.6)	0.09	4.9 (3.2)	5.0 (3.5)	0.96	7.9 (3.0)	8.2 (2.9)	0.68	7.5 (3.3)	7.8 (3.2)	0.50
No	4.3 (3.2)	5.0 (3.5)	0.005*	4.2 (2.9)	4.9 (3.3)	0.006*	7.8 (3.1)	8.1 (2.8)	0.25	7.8 (2.9)	7.7 (2.8)	0.24
Parents social support												
< = 54	4.8 (3.2)	5.4 (3.7)	0.001*	4.7 (3.1)	5.0 (3.4)	0.23	7.5 (3.1)	8.2 (3.2)	0.009*	7.8 (3.3)	8.2 (3.3)	0.16
> 54	3.6 (2.8)	4.5 (3.2)	< 0.001*	3.4 (2.7)	4.2 (3.2)	0.001*	7.4 (3.5)	7.4 (3.1)	0.83	7.2 (3.1)	7.5 (2.9)	0.24
Teachers social support												
Low < = 50	4.5 (3.2)	5.1 (3.5)	0.001*	4.6 (3.1)	4.7 (3.4)	0.42	7.6 (2.9)	7.7 (3.2)	0.59	8.0 (3.2)	7.9 (3.4)	0.76
High > 50	3.8 (2.9)	4.8 (3.4)	< 0.001*	3.7 (2.8)	4.5 (3.3)	< 0.001*	7.4 (3.6)	7.8 (3.1)	0.055	7.0 (3.1)	7.7 (2.8)	0.017*
Aggression Questionnaire Refined												
Low < = 27	3.3 (2.6)	4.1 (3.0)	< 0.001*	3.1 (2.4)	4.1 (3.1)	< 0.001*	6.4 (3.4)	6.6 (3.3)	0.34	6.7 (3.0)	7.1 (3.0)	0.11
High > 27	5.2 (3.3)	6.0 (3.6)	< 0.001*	5.0 (3.1)	5.1 (3.5)	0.83	8.3 (3.0)	8.6 (2.7)	0.18	8.2 (3.2)	8.5 (3.0)	0.27
Empathy												
Low < = 8	4.3 (3.0)	4.6 (3.4)	0.16	4.3 (2.9)	5.1 (3.2)	0.001*	7.5 (3.2)	7.8 (3.1)	0.34	7.9 (3.3)	7.9 (3.3)	0.95
High > 8	4.1 (3.2)	5.1 (3.5)	< 0.001*	4.0 (3.0)	4.3 (3.4)	0.10	7.4 (3.4)	7.7 (3.3)	0.18	7.1 (3.0)	7.7 (2.9)	0.017*
Total	4.18 (3.08)	4.96 (3.44)	< 0.001*	4.11 (2.94)	4.62 (3.34)	0.001*	7.45 (3.30)	7.74 (3.16)	0.102	7.42 (3.16)	7.78 (3.07)	0.073

^*^p < 0.05

**Table 4:** Intervention's Effect on Machismo and Acceptance of Violence in Girls and Boys

	Machismo		Acceptance of Violence					
	**Girls**		**Boys**		**Girls**		**Boys**	
	β	*p*-value	β	*p*-value	β	*p*-value	β	*p*-value
**Age (unexposed group: < = 13)**								
> 13 years	0.089	0.111	0.092	0.176	0.001	0.985	0.004	0.947
**Mother' education (Unexposed group: primary studies)**								
Secondary/superior	-0.041	0.328	-0.002	0.975	-0.026	0.542	0.010	0.838
**Dating violence (unexposed group: “I have never been in a dating relationship”)**								
I have been in a dating relationship, and I have been a victim of IPV	0.050	0.205	-0.025	0.612	-0.021	0.597	-0.035	0.465
I have been in a dating relationship, but I have not been a victim of IPV	-0.014	0.711	0.030	0.538	-0.001	0.974	-0.020	0.689
**Parents social support (unexposed group: scores < = 54)**								
Scores > 54	-0.025	0.540	-0.098	0.042*	-0.027	0.519	-0.083	0.076
**Teachers social support (unexposed group: scores < = 50)**								
Scores > 50	-0.001	0.979	0.065	0.165	-0.003	0.948	0.071	0.123
**Aggressiveness at baseline (unexposed group: scores < = 27)**								
Scores > 27	0.074	0.070	0.146	0.002*	-0.009	0.824	0.127	0.005*
**MVQ (machismo or AV) in baseline**	-0.488	< 0.001*	-0.509	< 0.001*	-0.354	< 0.001*	-0.524	< 0.001*
**Empathy in baseline group (unexposed group: scores < = 8)**								
Scores > 8	-0.406	0.251	0.120	0.056	0.110	0.029*	0.039	0.364
**Group (unexposed group: control group)**								
Intervention group	0.039	0.283	0.247	< 0.001*	0.077	0.207	0.010	0.814
**Interaction empathy x group**			-0.194	0.018*	-0.193	0.006*		

^*^p < 0.05

$${Y}_{i2}-{Y}_{i1}= {\beta }_{0}+ {\beta }_{1}{Y}_{i1}+\cdots + {\varepsilon }_{i}$$

$$\beta_0\;\mathrm{and}\;\beta_1=coefficients\;of\;the\;model;\;\varepsilon{\mathrm i}=random\;errors$$

Equation 1. Equation to obtain the difference in the value on wave-2 and wave-1.

To analyze whether the intervention had a different effect for each of the categories of covariables included in the model, we explored the interactions between the group variable (control vs. intervention) and each covariable. The results were stratified by sex. The statistical program STATA 14.0 was used for the analysis.

## Results

We collected 1,555 questionnaires in wave-1 (pre-test) and 1,434 questionnaires in wave-2 (post-test). There were 1,155 paired questionnaires from wave-1 and wave-2. Of these, nine questionnaires were excluded due to lack of response in the sex variable. The final data included 1,146 questionnaires: 575 for the intervention group (59.1% girls) and 571 for the control group (62.7% girls). The sociodemographic characteristics and average values of the scales used at baseline (wave-1) are presented in Table 1.

For girls at baseline, statistically significant differences between the control and intervention groups were identified by age, mother’s education, SS of teachers and exposure to IPV. A higher percentage of girls over the age of 13 belonged to the control group. In terms of mother’s education level, control group participants reported further education more frequently than the intervention group. The intervention group reported greater SS from teachers than the control group. Girls in the intervention group experienced more IPV than those in the control group. In terms of boys, differences between the control and intervention groups were only found for age.

### Has the intervention changed the levels of machismo and acceptance of violence in boys and/or girls?

The unadjusted mean values for machismo (Control group: Machismo_W1:_ 10.94; Machismo_W2_: 9.92. Intervention group: Machismo_W1_: 10.02; Machismo_W2_: 10.31) and AV (Control group: AV_W1:_ 7.45; AV_W2_: 7.74. Intervention group: AV_W1_: 7.42; AV_W2_: 7.78) were greater in boys, both in the control group and in the intervention group. However, the differences between wave-1 and wave-2 were only statistically significant for AV in girls, both in the control group and the intervention group (*p*-value=<0.001, effect size_control: 0.239; *p*-value=0.001, effect size_intervention: 0,162). For boys differences between wave-1 and wave-2 were only detected for machismo in control group (*p*-value=0.036, effect size_control: -0.125).

Table 2 shows that mean values for machismo in wave-1 and wave-2 by group (control/intervention) and by sex, for each of the covariables. In girls, for the intervention and control groups, there were no changes observed for any of the variables in wave-2, compared to wave-1. In boys in the intervention group, there was a significant increase in the mean values of machismo in wave-2, compared to wave-1, among those who perceived high SS from teachers. In boys, for the control group there was a significant decrease in the mean values of machismo in wave-2, compared to wave-1, in those who had experienced IPV and who perceived a high SS from fathers/mothers, and who had low empathy (*p*<0.05) in wave-1.

Table 3 shows the mean values of AV in wave-1 and wave-2 by group (control/intervention) and by sex, for each of the covariables. In girls in the intervention and control group there was a significant increase in the average values of AV for nearly all the variables. Those boys from the intervention group who have never been in a dating relationship (*p*<0.001) perceived high SS from teachers (*p*<0.05) and had high levels of empathy (*p*<0.05), scored higher on AV in wave-2 compared to wave-1. In the control group there was an increase in the average values of AV among those who perceived a low level of support from parents (*p*<0.01).

### What influence do baseline scores in empathy, aggressiveness and/or social support have on intervention’s result?

**Fig. 1:**
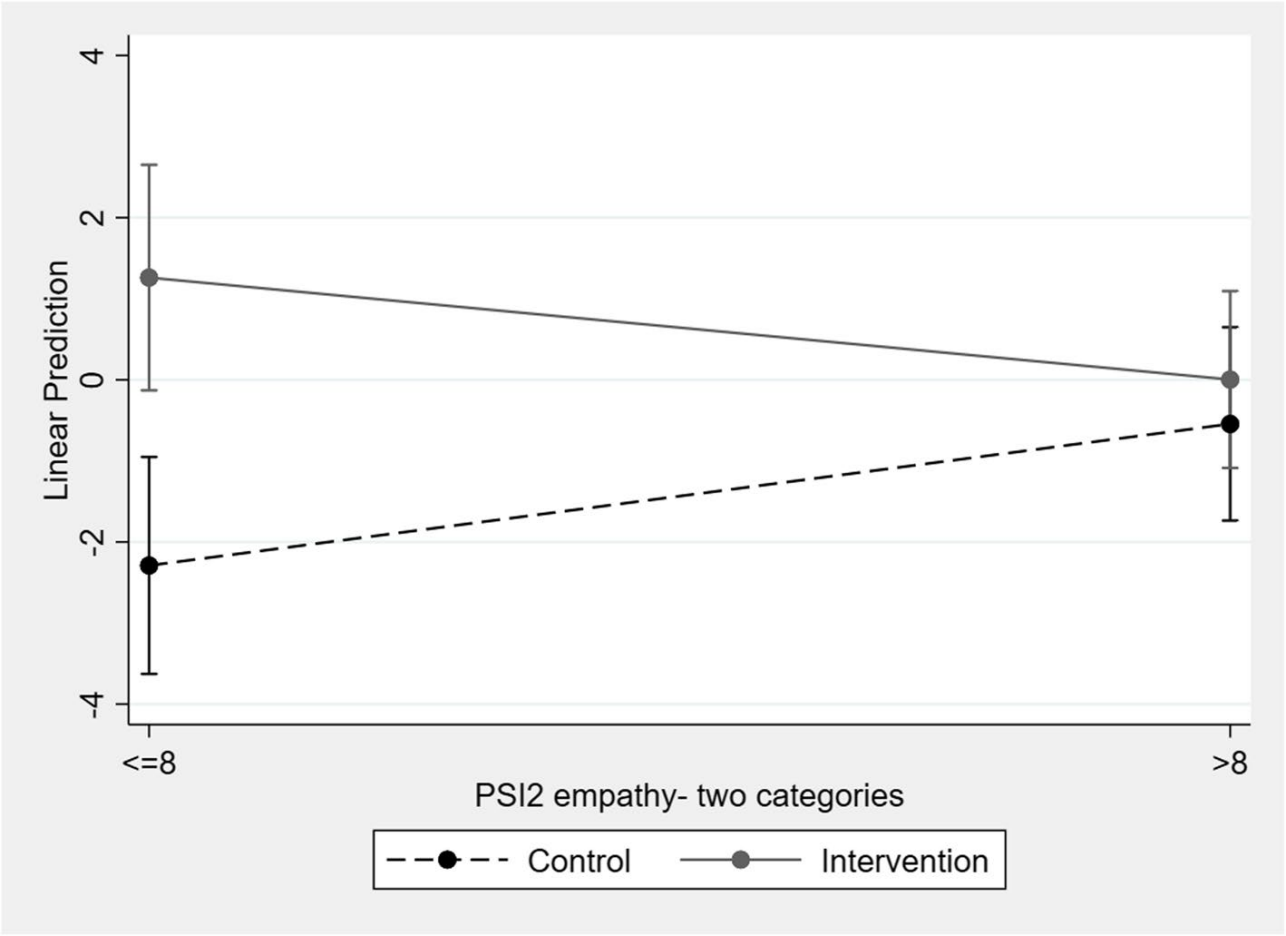
Interaction between empathy and group in Machismo (boys)

Table 4 shows the variables associated with the change in values of machismo and AV over time (wave-2 - wave-1). In modeling the change in machismo, an interaction was identified between the group variable and the empathy variable in the sample of boys (*p*=0,0018) (see Fig. 1). Among boys from the intervention group that showed low baseline empathy (reference: control group), a significant increase was observed in the mean values of machismo (β:0.247; *p*<0.001).

In the same way, those who had higher average scores on SS from fathers/mothers at baseline decreased in terms of levels of machismo in wave-2 (β: -0,098; *p*=0,042), both in the intervention group and in the control group. Boys in both the intervention group and the control group who had higher average scores on aggressiveness at baseline had increased average scores on machismo in wave-2 (β: 0,146; *p*=0,002).

**Fig. 2:**
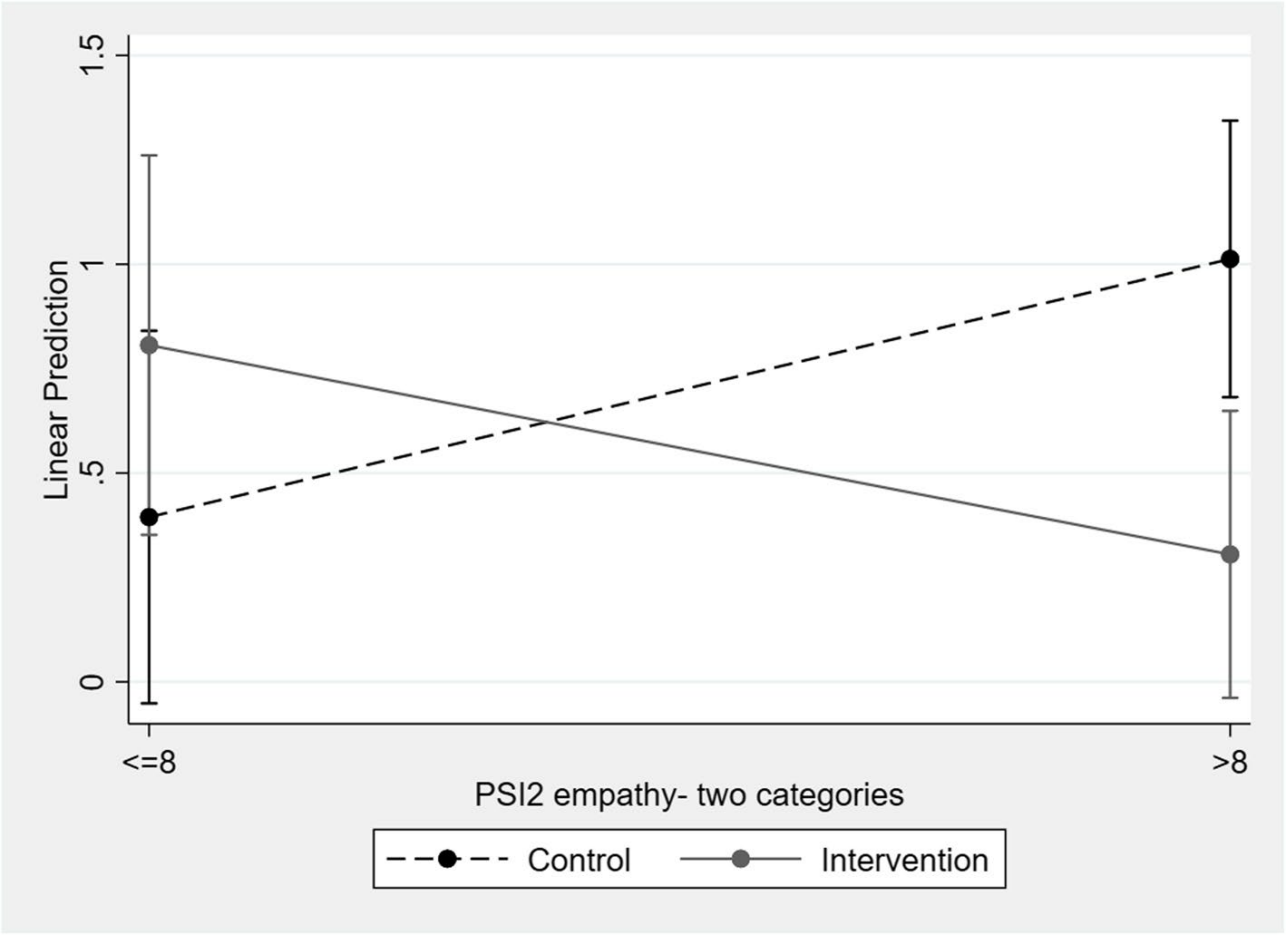
Interaction between empathy and group in Acceptance of Violence (girls)

In terms of the change in AV, an interaction was identified between the group variable and the empathy variable in the sample of girls (*p*=0.006) (see Fig. 2). Compared to the control group, the girls in the intervention group who showed high empathy at baseline obtained lower average scores on AV (β:-0,131; *p*=0,004).

In boys, those who had higher average scores on aggressivity (at baseline) increased in average scores for AV in wave-2, both in the intervention group and in the control group (β:0,127; *p*=0,005).

For both sexes the change in average values of machismo and AV was associated with corresponding values obtained at baseline. The high average baseline values in machismo (wave-1) were associated with a greater reduction in machismo in wave-2. The same occurred for AV. This effect was independent of the group of participants (intervention or control).

## Discussion

Boys in the study reported higher mean values for machismo and AV than the girls. The effectiveness of the intervention varied by sex and by prior level of empathy of the participants. The boys in the intervention group with low empathy at the start of the program scored high on machismo after the intervention, compared to the control group. The girls who initially scored high on empathy had lower average levels of AV after the intervention, compared to the control group. Furthermore, we observed that among boys who had initially scored high average values on SS from fathers/mothers, there was a decrease in machismo in the second wave, although this decrease was present in both groups (intervention/control). On the contrary, the boys in the intervention and control groups that scored high on aggressiveness at the beginning of the study had higher average scores on machismo during the second wave.

The fact that macho attitudes and acceptance of violence are more common in boys has also been demonstrated in other studies [8]. This could be explained by the differences in the socialization process of boys and girls, which involve different gender roles [27], in which traditional masculine norms include suppressing emotion and emotional dysregulation. Macho attitudes are the result of the construction of gender roles and norms (related to hegemonic masculinity), and these attitudes sustain a culture of violence among boys [28].

Educational interventions are more effective in terms of violent behavior when they focus on improving social abilities (such as empathy), instead of changing thought patterns or focusing on the consequences [29]. In this study, empathy played an important role in terms of the effectiveness of the Lights4Violence in reducing machismo and acceptance of violence. Specifically, we observed that the boys with low empathy obtained higher scores on machismo after the intervention, compared to the control group, as has occurred in prior studies [17, 30]. Macho attitudes reflect hegemonic masculinity. Development of active listening and emotional management skills is uncommon in boys who have been educated in a culture that associates masculinity with rationality, emotional control, and limited capacity for collaboration [31].

As has been shown before [32], the girls in the intervention group with greater empathy at the beginning of the study scored lower on AV in the second wave, compared to the control group. Gender roles play an important role at this point too. The models established by Blair [33] suggested that empathy facilitates moral socialization, concluding that the people with more empathy are more motivated and could be more susceptible to inhibit violence that may be the case of our targeted girls.

Furthermore, external health assets, such as SS from fathers/mothers, are considered protective factors against the perpetration of violence [34]. In this study, the boys who had a good relationship with their parents at the beginning of the study scored lower average scores on machismo, both in the control group and in the intervention group. We are not aware of other studies with the same results. However, some studies have found a relationship between parenting style and sexist attitudes, with a greater impact on sons than on daughters [35]. Fathers/mothers had an important role in gender socialization of adolescents (development of gender roles and norms) [36], and this could influence the response to a situation. This could explain why the control group decreased in terms of its levels of machismo and AV, despite not having received the intervention.

Boys with higher levels of aggressiveness at baseline increased in terms of values of machismo and AV in the second wave, independently of the intervention. To our knowledge, there are no other interventions in the literature with the same results. However, there have been studies of the association between aggressiveness and violence and predisposing factors (the first is a precursor to the latter) [29]. One possible explanation could be related to the Hawthorne Effect, which refers to participation in research and the consequent awareness of being studied and a possible impact on behaviour [37]. It could be that boys had a reaction to the questionnaire in the second wave and perceived that their masculinity was being “questioned” (they may have felt controlled by others) [38]. This could be a possible explanation for the fact that boys show greater resistance to change in these types of interventions, feeling “observed” or “singled out”, with an effect contrary to what would be expected [31]. In addition, findings from studies examining the reaction of anti-violence campaigns and interventions have found the same pattern of results, suggesting that individuals with higher scores in trait aggressiveness react more favourably toward violence [39]. Some authors have suggested that these individuals are much more vulnerable because they perceive as threatening the messages of anti-violence campaigns making them feel bad about their behaviour [40].

These concerning results may be also due to the influence of moral disengagement in aggression, which is a psychological process by which people use “buffers” between their moral principles and how they really behave [41]. This mechanism is useful in legitimizing behaviours or attitudes considered violent (or contrary to social morality), and in understanding aggressive behaviour as a tool to pursue and achieve personal goals [42]. This psychological process has been studied in both DV perpetration and victimization [43]. Sexual assaults that occur in dating relationships have been shown to be associated with lower levels of guilt and shame [44]. Therefore, mental strategies such as moral disengagement may also have an influence when it comes to changing certain attitudes such as machismo or AV in those who have high scores in baseline aggressiveness. In addition, this mechanism has been negatively related to empathy [45], which would also explain that in the intervention group, those boys who had low empathy at baseline have higher scores in machismo, while girls with high empathy had a decrease in their AV scores. Likewise, in the “results” section, two figures have been added about the interaction between empathy and group for machismo (boys) and AV (girls) to clarify the results obtained.

### Limitations

This study should be interpreted considering several limitations. The sample was selected via a non-probabilistic method. The schools were selected for reasons of viability. The distribution of the schools in the intervention and/or control group was not carried out randomly. However, the differences identified between the intervention group and the control group were introduced into the statistical models to avoid spurious associations. The follow-up time after the intervention was limited in terms of evaluation of participants, thus it is unknown whether the effect of the intervention on machismo and AV was maintained. In the same way, it is possible that the intervention produced effects on machismo and AV over time that are not observed in this study. It was not possible to analyze the characteristics of the lost sample due to the difficulty in identification of coding.

Empathy was evaluated through three items belonging to another scale that assess es personal strengths related to socio-emotional competencies [22]. Although the Cronbach’s alpha obtained for these three items was good (0.680), one limitation of the scale involves the difficulty each person might face in interpreting and understanding what other people are feeling, thinking and when they are upset.

It would have been interesting to include variables related to sexual orientation. However, it was not possible due to sensitivity of the question in certain countries.

## Conclusions

Despite the limitations described above, it is worth highlighting that empathy is important in the effectiveness of educational interventions with adolescents. While Lights4Violence focuses on the promotion of assets for developing healthy couple relationships, there have been some controversial results. Perhaps some boys, especially those with less empathy, felt “questioned” during the intervention and/or evaluation. On one hand, it is important to consider the psychological processes that could influence the development of personal skills, because they may make it difficult to change certain attitudes. On the other hand, it is necessary to develop interventions that specifically approach hegemonic masculinity to break with legitimization of violence and to promote alternative expressions of masculinity that help combat violence of men against women [46].

## Data Availability

The datasets and material are available from the corresponding author on reasonable request that guarantee their use according to the ethical procedures adopted in this project and the contents of participants’ informed consent.

## Abbreviations

AV: Acceptance of Violence
IPV: Intimate Partner Violence
SS: Social Support
MVQ: Maudsley Violence Questionnaire
CASSS: Child and Adolescent Social Support Scale
